# The Identification of Opioid Receptors and Peptide Precursors in Human DRG Neurons Expressing Pain-Signaling Molecules Confirms Their Potential as Analgesic Targets

**DOI:** 10.3390/cells14100694

**Published:** 2025-05-11

**Authors:** Shaaban A. Mousa, Mohammed Shaqura, Sascha Tafelski, Jan David Wandrey, Özgür Celik, Sascha Treskatsch, Michael Schäfer

**Affiliations:** 1 Charité–Universitätsmedizin Berlin, Corporate Member of Freie Universität Berlin, Humboldt-Universität zu Berlin, and Berlin Institute of Health, Department of Anaesthesiology and Intensive Care Medicine, Charité Campus Benjamin Franklin, Hindenburgdamm 30, 12203 Berlin, Germany; mohammed.shaqura@charite.de (M.S.); ozgur.celik@charite.de (Ö.C.); sascha.treskatsch@charite.de (S.T.);; 2 Charité–Universitätsmedizin Berlin, Corporate Member of Freie Universität Berlin, Humboldt-Universität zu Berlin, and Berlin Institute of Health, Department of Anaesthesiology and Intensive Care Medicine, Charité Campus Mitte and Campus Virchow Clinic, Charitéplatz 1, 10117 Berlin, Germany; sascha.tafelski@charite.de (S.T.); jan.wandrey@charite.de (J.D.W.); 3 Berlin Institute of Health at Charité–Universitätsmedizin Berlin, BIH Biomedical Innovation Academy, BIH Charité Digital Clinician Scientist Program, Charitéplatz 1, 10117 Berlin, Germany

**Keywords:** opioid receptors, opioid peptides, peripheral sensory neurons, pain, human

## Abstract

The presence and function of the opioidergic system in sensory dorsal root ganglia (DRG) was demonstrated in various animal models of pain. To endorse recent functional and transcriptional evidence of opioid receptors in human DRG, this study compared morphological and transcriptional evidence in human and rat DRG using immunofluorescence confocal microscopy and mRNA transcript analysis. Specifically, it examined the neuronal expression of mu (MOR), delta (DOR), and kappa (KOR) opioid receptors, opioid peptide precursors (POMC, PENK, and PDYN), and key pain-signaling molecules. The results demonstrate abundant immunoreactivity in human DRG for key pain transduction receptors, including the thermosensitive ion channels TRPV1, TRPV4 and TRPA1, mechanosensitive PIEZO1 and PIEZO2, and the nociceptive-specific Nav1.8. They colocalized with calcitonin gene-related peptide (CGRP), a marker for peptidergic sensory neurons. Within this same subpopulation, we identified MOR, DOR, and KOR, while their ligand precursors were less abundant. Notably, the mRNA transcripts of MOR and PENK in human DRG were highest among the opioid-related genes; however, they were considerably lower than those of key pain-signaling molecules. These findings were corroborated by functional evidence in demonstrating the fentanyl-induced inhibition of voltage-gated calcium currents in rat DRG, which was antagonized by naloxone. The immunohistochemical and transcriptional demonstration of opioid receptors and their endogenous ligands in both human and rat DRG support recent electrophysiologic and in situ hybridization evidence in human DRG and confirms their potential as analgesic targets. This peripherally targeted approach has the advantage of mitigating central opioid-related side effects, endorsing the potential of future translational pain research from rodent models to humans.

## 1. Introduction

The activation of opioid receptors (ORs) within the central nervous system (CNS) produces potent analgesia along the pain transmission pathway [1,2]. Three main opioid receptors—MOR, DOR, and KOR—have been pharmacologically characterized, and their endogenous ligands and precursor peptides—POMC, PENK, and PDYN—have been identified, all of which were localized within the brain and spinal cord of mammals [3], To date, only MOR agonists have found their way into clinical practice. While they are considered to be the most potent analgesics, their use is associated with significant central side effects and a high potential for abuse. Beyond their presence in the CNS, ORs have also been identified on peripheral sensory neurons, particularly in rodents such as rats and mice [4]. Importantly, preclinical studies have demonstrated that activating peripheral opioid receptors on the neurons of dorsal root ganglia (DRG) produces analgesic and anti-inflammatory effects in rodent models without inducing centrally mediated side effects [4,5]. This discovery led to the first clinical report demonstrating the analgesic efficacy of intra-articular morphine application following arthroscopic surgery of a knee trauma [6]. However, the clinical relevance of peripheral opioid analgesia remains a topic of debate [7,8,9]. In response to these concerns, a new class of peripherally restricted opioid analgesics has been developed, aiming to maximize pain relief while minimizing central opioid-related side effects. These drugs do not cross the blood–brain barrier and specifically target opioid receptors on the peripheral sensory neurons of inflamed or traumatized tissue [10,11,12,13]. Although preclinical results from these newly developed drugs are promising, they have not yet found their way into clinical practice.

A growing body of experimental evidence highlights DRG neurons as a key player in pain sensation and signal transmission to the CNS [14,15]. This was recently demonstrated by thorough investigation of key pain-signaling molecules using RNAscope in situ hybridization [15,16,17,18,19]. Most interestingly, the authors compared pain-relevant transcriptomes and mRNA profiles between rodent and human DRG. While morphological and genetic similarities between humans and rodents are well documented, critical differences exist. Despite increasing knowledge of the opioidergic system in the rodent peripheral nervous system [4], translating somatosensory mechanisms from experimental models to clinical applications remains challenging due to molecular differences between peripheral sensory neurons in rodents and humans.

Recently, several outstanding studies on human DRG have begun to address this gap [20,21,22], providing electrophysiological and transcriptional evidence for the presence of opioid receptors through patch-clamp recordings and multiplex in situ hybridization. The primary aim of our study, however, was to further support these findings with immunohistochemical evidence of the three main opioid receptors—mu (MOR), delta (DOR), and kappa (KOR)—as well as the opioid peptide precursors (POMC, PENK, and PDYN), alongside key pain-signaling molecules. We compared their immunoreactivity between human and rat dorsal root ganglia (DRG) and further substantiated our observations using quantitative real-time PCR and functional electrophysiological experiments.

## 2. Materials and Methods

### 2.1. Collection of Human and Rat DRG Tissue Samples

Frozen human DRG (L4, right and left) from two young fatal-accident victims (one female, one male) were commercially obtained from AnaBios Corporation and Donor Network West (San Roman, CA, USA) following US authority approval. The DRG supplier obtained the appropriate informed consent of all subjects contributing biological materials, and all other authorizations, consents, or permissions necessary for the transfer and use of the biological materials for research. The DRG tissue samples stained with hematoxylin–eosin were assessed for morphological tissue integrity prior to downstream applications; then, they were immediately stored in liquid nitrogen (n = 2), and they underwent tissue fixation by being subjected to 4% paraformaldehyde (n = 2) prior to shipment. The frozen tissue DRG samples were divided in half to obtain two technical replicates and used for quantitative real-time PCR (see below for more details). In addition, paraformaldehyde-fixed tissue samples were used for immunohistochemistry (see below for more details). Following IRB approval by the LaGeSo Berlin, rat lumbar DRG (L3–5) were removed from untreated normal Wistar rats (250–300 g body weight) and processed as fresh frozen samples for quantitative real-time PCR (n = 4–5 rats) or were paraformaldehyde-fixed (n = 5 rats) for subsequent immunohistochemistry.

### 2.2. Quantitative qRT-PCR in Human and Rat DRG Neurons

Gene expression analysis was performed using real-time PCR as previously described [23]. Total RNA was extracted from human and rat DRG and a total of 1000 ng of RNA was transcribed into cDNA using the Omniscript RT Kit (Qiagen) as follows: 0.5 mM dNTP, 5 µM random primer, 10 U RNase inhibitor, and 4 U Omniscript reverse transcriptase [23]. Samples were incubated at 42 °C for 1 h, and cDNA was stored at −20 °C afterwards. Primers were carefully validated by carrying out a melting curve analysis and a standard curve generation [23]. The following specific primer pairs for human (see Supplementary Table S1) and rat (see Supplementary Table S2) MOR, DOR, KOR, POMC, PENK, PDYN, TRPV1, TRPV2, TRPV4, TRPA1, TRPM8, PEZO1, PIEZO2, Nav.1.8, Nav1.9, and 18S as the housekeeping gene were used. Real-time PCR was performed with the Eppendorf Vapo Protect Mastercycler Pro^®^ using a SYBR Green kit following the manufacturer’s instructions (Eppendorff AG, Hamburg, Germany). Amplification was carried out for 40 cycles, each consisting of 15 s at 95 °C for all genes except 18S, where each cycle consisted of 30 s at 60 °C [23]. A temperature of just below the specific melting temperature was employed in the PCR for detecting fluorescence-specific products [23]. The housekeeping gene 18S rRNA was used as an internal reference gene for quantification. Samples were measured in triplicate and the evaluation was performed according to the ΔΔCT method, i.e., ΔCt values were obtained by Ct_gene_–Ct_18S_ housekeeping gene and subsequently related to ΔCt values of MOR [24].

### 2.3. Immunohistochemistry in Human and Rat DRG

All tissue preparations followed established protocols described in previous studies [23]. Human DRG samples were pre-fixed in 4% paraformaldehyde and underwent an extended water wash for 48 h. Rat DRG tissues were obtained from naïve rats (Janvier Labs) following deep isoflurane anesthesia. Once anesthetized, the rats were transcardially perfused with 100 mL of warm saline, followed by 300 mL of 4% (*w*/*v*) paraformaldehyde in 0.16 M phosphate buffer (pH 7.4). After perfusion, DRG were harvested and post-fixed in the same fixative for 90 min [23]. Both human and rat DRG tissues were cryoprotected overnight at 4 °C in PBS containing 10% sucrose. Samples were then rinsed with PBS and stored at −80 °C for further processing. DRG sections, cut to a thickness of 10 μm, were mounted onto gelatin-coated slides. Immunostaining was performed on every fourth section of serially cut DRG (10 μm) from each rat (n = 5) and human donor (n = 2), with a minimum of 10 sections stained per antibody.

Slide-mounted tissue sections were washed with PBS, then incubated with ice-cooled freshly prepared sodium borohydride (1 mg/mL in PBS) for three consecutive 10 min intervals before the blocking step commenced. Slide-mounted tissue sections were washed in PBS and incubated with a blocking solution (PBS containing 0.3% Triton X-100, 1% BSA, 10% goat serum, 10% donkey serum) for 60 min. Tissue sections were then incubated overnight with the following primary antibodies against MOR, DOR, KOR, POMC, PENK, PDYN, TRPV1, TRPV4, TRPA1, PEZO1, PIEZO2, and Nav.1.8 (see details in Supplementary Table S3) alone or in combination with another antibody for double immunofluorescence. After incubation with the primary antibodies, the tissue sections were washed with PBS and incubated with Alexa Fluor 594 donkey anti-rabbit antibody (Vector Laboratories) in combination with Alexa Fluor 488 goat anti-guinea-pig, or anti-mouse antibody (Invitrogen, Schwerte, Germany). Thereafter, sections were washed with PBS, and the nuclei were stained bright blue with 4′-6-Diamidino-2-phenylindole (DAPI) (0.1 μg/mL in PBS) (Sigma). Finally, tissues were washed in PBS, mounted in vectashield (Vector Laboratories), and imaged on a confocal laser scanning microscope, namely Zeiss LSM510, equipped with an argon laser (458/488/514 nm), a green helium/neon laser (543 nm), and a red helium/neon laser (633 nm) (Carl Zeiss, Göttingen, Germany), as described previously [25]. The settings for contrast, brightness, scanning time, and pin hole were identical for each picture in each individual set. The fluorescence emission was recorded through a PLAN-NEOFLUAR 40, NA 1.3 oil objective (Zeiss; Oberkochen, Germany). The quantification of DRG staining has been described previously [25,26]. Quantification of the immunofluorescence of MOR, DOR, and KOR/CGRP, as well as MOR/Nav1.8, in human DRG tissue sections was performed by using the Zeiss Zen 2009 software Carl Zeiss Micro-Imaging GmbH (Göttingen, Germany). Values were presented as median [25th;75th] percentages. To demonstrate the specificity of staining, we performed the following endogenous tissue controls (no primary or secondary antibody) and primary antibody controls (just secondary antibody) to reveal the level of autofluorescence and non-specific binding in our immunofluorescence experiments, as described in previous studies [25,26]. As we have outlined previously [25,26], the specificity of the anti-DOR antibody was demonstrated by in vitro and in vivo antisense and knock-out experiments [27]; the specificity of the anti-MOR antibody was demonstrated by MOR-transfected CHO-K1 cells and HEK-293 cells and by in situ hybridization experiments [28], whereas the specificity of the anti-KOR antibody was demonstrated by KOR-transfected COS-1 cells in comparison to MOR- and DOR-transfected COS-1 cells [25,26,29] (see also Supplemental Figure S1). These three antibodies have been extensively and successfully used in rodents [25,26] and humans [30,31]. Therefore, these antibodies were sufficiently validated. Similar specificity has been shown for the other antibodies, e.g., the specificity of the polyclonal anti-PIEZO2 antibody was tested by preincubating it with the manufacturer’s specific PIEZO2 antigenic peptides (2 mg/mL) for 2 h before immunostaining, as described previously [32].

### 2.4. Electrophysiological Experiments of Opioids in Rat DRG

Patch-clamp experiments were performed on dorsal root ganglia (DRG) neurons extracted from male Wistar rats (n = 10). The neurons (n = 16–20, Ø between 20 and 30 µm) were studied 24–48 h after dissection, using a modified version of a protocol published in 2007 [33]. Prior to each experiment, cell viability was confirmed using an automated cell counter (Luna, Villeneuve, France) with acridine orange and propidium iodide staining. During the recordings, the cells were kept in extracellular solution (ECS) and visualized with a Zeiss Axiovert 200 inverted microscope (Zeiss, Jena, Germany). Recording pipettes, with a resistance of 3.5–8 MΩ, were created on-site from borosilicate glass capillaries (Harvard Bioscience, MA, USA) using a Sutter P-97 puller (Sutter Instruments, Novato, CA, USA). These pipettes were filled with intracellular solution (ICS) before use. Electrical currents were measured using an EPC-10 patch-clamp amplifier and recorded with Pulse software v8.66 (HEKA, Lambrecht, Germany). The ECS was continuously delivered at a rate of 800–1000 µL/min via a pressurized perfusion system (Perfusion Pressure Kit VPP-6; Warner Instruments, Hamden, CT, USA). Only neurons with diameters of between 20 and 30 µm were selected for these recordings. After reaching a “giga-seal” at −60 mV, the membrane patch was ruptured to establish the whole-cell configuration. Initial currents were recorded at a holding potential of −80 mV in ECS buffer without test compounds. Cells were then depolarized to +10 mV for 100 ms, and this was repeated eight times at 20 s intervals. ECS alone was perfused for the first five cycles. On the sixth cycle, fentanyl (a MOR agonist) was introduced. To confirm the opioid-receptor-mediated effects, naloxone (a MOR antagonist) was added during the subsequent cycle. Fentanyl and naloxone were administered via the perfusion valve system VC6. All recordings were performed at room temperature. The effects were quantified by measuring the percentage reduction in current amplitude relative to the control conditions (ECS alone). Sample size was determined using G*Power 3.1.2 (α < 0.05, power = 80%), based on pilot experiment effect size. To minimize bias, experiments were randomized and blinded. Data and statistical analyses were performed using Prism (GraphPad, USA). Grubbs’ test was used to assess outliers, and none were identified. Normality and variance were assessed using the D’Agostino and Pearson test. Concentration–response relationships were analyzed by simple linear regression. Each experiment included 16–20 DRG neurons. Statistical significance was determined using a two-tailed *t*-test.

### 2.5. Statistics

Statistical analyses were performed using the Sigma Stat 2.03 software (SPSS, IBM Inc., Armonk, NY, USA). Descriptive statistics were used to present the data as means ± standard deviation. Quantitative qRT-PCR experiments were intended to be purely exploratory and were analyzed by the nonparametric Kruskal–Wallis-test followed by a post hoc Dunnett’s method. For the statistical analyses of the electrophysiological experiments, see the respective paragraph above.

## 3. Results

### 3.1. Identification of Key Pain-Signaling Molecules in CGRP-IR Sensory Neurons of Human Compared to Rat DRG Neurons

**Figure 1:**
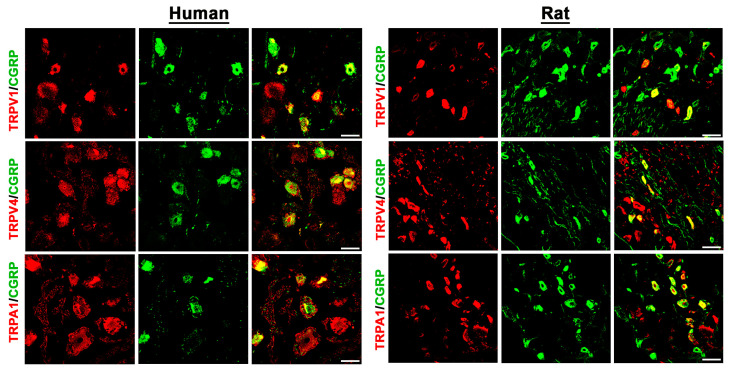
Detection of the heat-sensitive TRPV1, TRPV4, and cold-sensitive TRPA1 pain transduction receptors in human versus rat sensory DRG neurons. TRPV1, TRPV4, and TRPA1 were visualized by specific antibodies (see Supplementary Table S3) and respective secondary antibodies labelled with Texas red fluorescence, while the sensory neuron marker CGRP was visualized by FITC green fluorescence. Both in human (**left panel**) as well as in rat (**right panel**) DRG neurons, TRPV1, TRPV4, and TRPA1 immunoreactivity is clearly detectable and colocalizes (yellow fluorescence) abundantly with the sensory neuron marker CGRP. Bars represent 55 µm.

**Figure 2:**
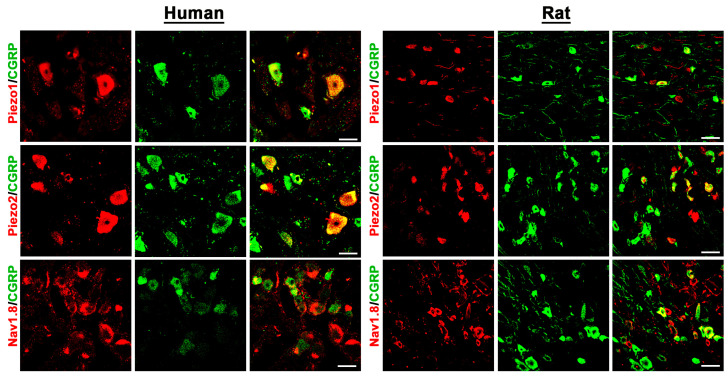
Detection of the mechanosensitive ion channels PIEZO1 and PIEZO2, as well as the TTX-resistant voltage-gated Nav1.8 ion channel, in human versus rat sensory DRG neurons. PIEZO1, PIEZO2, and Nav1.8 were visualized by specific antibodies (see Supplementary Table S3) and respective secondary antibodies labelled with Texas red fluorescence, while the sensory neuron marker CGRP was visualized by FITC green fluorescence. Both in human (**left panel**) as well as in rat (**right panel**) DRG neurons, PIEZO1, PIEZO2, and Nav1.8 immunoreactivity is clearly detectable and colocalizes (yellow fluorescence) in part with the sensory neuron marker CGRP. Bars represent 55 µm.

Double immunofluorescence confocal microscopy identified the existence of the TRP-ion channel members TRPV1, TRPV4, and TRPA1, the mechanosensitive ion channels PIEZO1 and PIEZO2, as well as the TTX-resistant voltage-gated Nav1.8 ion channel, in human DRG neurons (Figure 1 and Figure 2). Moreover, our results showed that TRPV1, TRPV4, and TRPA1, as well as PIEZO1, PIEZO2, and Nav1.8 immunoreactivity, colocalized with the peripheral sensory neuron marker CGRP in a subpopulation of human DRG neurons (Figure 1 and Figure 2). Intriguingly, these same pain-signaling molecules could be localized in the CGRP-immunoreactive (IR) subpopulation of rat DRG neurons (Figure 1 and Figure 2).

### 3.2. Opioid Receptor Detection of MOR, DOR, and KOR in CGRP-IR Sensory Neurons of Human Compared to Rat DRG Neurons

**Figure 3:**
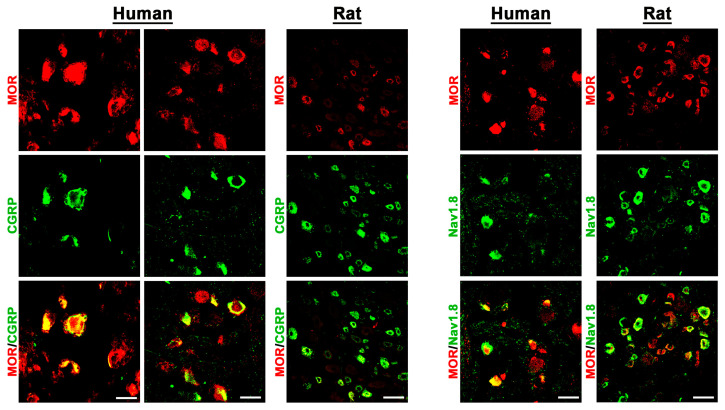
Colocalization of the opioid receptor MOR together with the peripheral sensory neuron marker CGRP or the nociceptive neuron marker Nav1.8 in human versus rat sensory DRG neurons. MOR was visualized by a specific antibody (see Supplementary Table S3) and respective secondary antibody labelled with Texas red fluorescence, while CGRP or Nav1.8 was visualized by FITC green fluorescence. Both in human (**left panel**) as well as in rat (**right panel**) DRG neurons, MOR immunoreactivity colocalized (yellow fluorescence) with CGRP or Nav1.8. Bars represent 55 µm.

**Figure 4:**
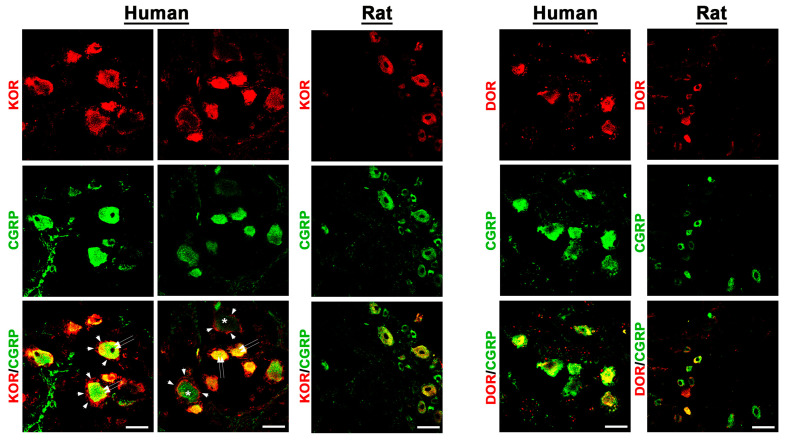
Detection of the opioid receptors KOR and DOR in human versus rat sensory DRG neurons. KOR and DOR were visualized by specific antibodies (see Supplementary Table S3) and respective secondary antibodies labelled with Texas red fluorescence, while the sensory neuron marker CGRP was visualized by FITC green fluorescence. Both in human (**left panel**) as well as in rat (**right panel**) DRG neurons, KOR and DOR immunoreactivity is clearly detectable and colocalizes (yellow fluorescence) abundantly with the sensory neuron marker CGRP (double arrow). Notably, KOR was also expressed in satellite glia cells (arrowhead) encircling KOR-positively or -negatively (*) stained DRG neuronal cell bodies. Bars represent 55 µm.

Using known specific antibodies [1,25] for the human MOR, DOR, and KOR, we could identify all three major opioid receptors in human DRG neurons, which were predominantly detectable in the subpopulation of CGRP-IR sensory neurons (Figure 3 and Figure 4).

In fact, preliminary counting of DRG tissue samples (n = 13–20) revealed that approximately 60%[51;66] of MOR neurons, 67%[61;73] of DOR neurons, and 67%[63;71] of KOR-IR DRG neurons colocalized with the sensory neuron marker CGRP, whereas 74%[60;82] of MOR-IR DRG neurons colocalized with the nociceptive neuronal marker Nav1.8. Notably, KOR immunoreactivity was also detectable in satellite glia cells (Figure 4). Some CGRP-immunoreactive neurons lacked MOR, DOR, or KOR immunoreactivity and vice versa. Moreover, it was demonstrated that MOR primarily colocalized with the TTX-resistant sodium channel Nav1.8, which is highly representative for peripheral nociceptive neurons (Figure 3).

### 3.3. Identification of the Opioid Peptide Precursors POMC, PENK, and PDYN in Human Compared to Rat DRG Neurons

**Figure 5:**
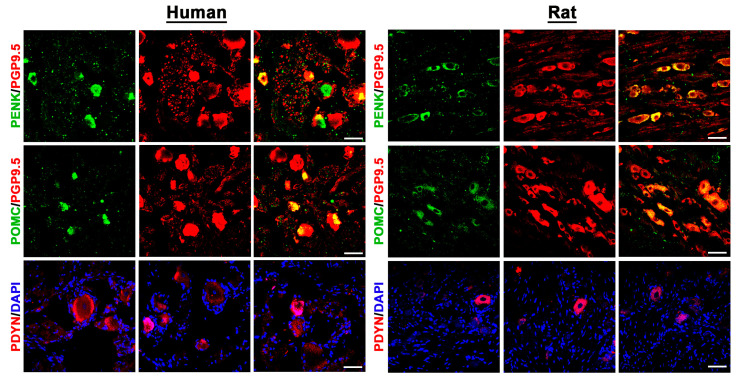
Detection of the opioid peptide precursors POMC, PENK, and PDYN in human versus rat sensory DRG neurons. POMC, PENK, and PDYN were visualized by specific antibodies (see Supplementary Table S3) and respective secondary antibodies labelled with FITC green fluorescence; because their neuronal cell staining was scarce, tissue sections were co-stained with the pan-neuronal marker PGP9.5 for all DRG neurons, visualized by Texas red fluorescence; since PDYN immunostaining was even less abundant, sections were counterstained only with the nuclear stain 4′-6-Diamidino-2-phenylindole (DAPI) for better visibility. The pictures clearly show that POMC or PENK immunoreactivity was exclusively visible in PGP9.5-positive DRG neurons (colocalisation, yellow fluorescence); also, notably, many PGP9.5 immunoreactive neurons lacked POMC or PENK immunoreactivity. Bars represent 55 µm.

Most interestingly, we also set out to identify the precursors of the endogenous opioid peptide ligands of these three opioid receptors. Our double immunofluorescence confocal microscopy showed specific immunoreactivity of the precursor peptides POMC and PENK colocalizing with the pan-neuronal marker PGP9.5 in some populations of human as well as rat DRG neurons (Figure 5).

However, PDYN immunoreactivity was scarce both in human and rat DRG neurons (Figure 5).

### 3.4. Predominant mRNA Expression of MOR over DOR and KOR, as Well as of the Endogenous Opioid Peptide Precursors, in Human Compared to Rat DRG Neurons

**Figure 6:**
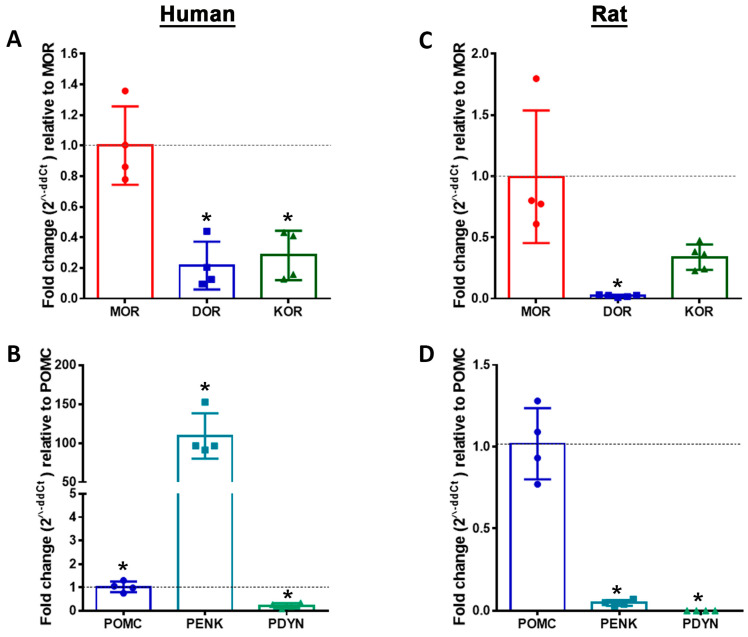
Detection of opioid receptor and opioid peptide precursor mRNA transcripts in human (**A**,**B**) versus rat (**C**,**D**) sensory DRG neurons. In using specific mRNA primers, quantitative real-time PCR showed a predominant mRNA expression of MOR over DOR and KOR in human DRG (5-fold), as well as MOR over DOR and KOR in rat (10-fold) sensory DRG neurons (**A**,**C**) (*p* < 0.05, Kruskal–Wallis test, followed by post hoc Dunn’s test). In addition, quantitative real-time PCR analyses of human DRG neurons revealed that PENK opioid peptide precursor mRNA was more abundant than POMC and PDYN mRNA, whereas POMC mRNA was superior in rat DRG neurons (**B**,**D**). Data are expressed as means ± SD. Statistical significance was calculated using the Kruskal–Wallis test, followed by post hoc Tukey’s test, with * *p* < 0.05.

Using highly specific primer pairs (see Table 1), opioid-receptor mRNAs for MOR, DOR, and KOR were detectable by qRT-PCR in both human and rat DRG with their respective Ct values (Ct value range: 25–30, see Table 1 (Figure 6).

Interestingly, MOR mRNA expression in human DRG was highest among all opioid receptors and was 5-fold higher than the expression of DOR and KOR mRNA (Figure 6). This was almost similar in rat DRG neurons, with the exception of KOR mRNA expression being higher than DOR mRNA.

Since, the different types of opioid receptors are activated by their respective endogenous ligands, we examined the expression of their respective precursors in human versus rat DRG. In parallel to the expression of all opioid receptors, the precursor POMC, PENK, and PDYN mRNAs of the corresponding endogenous opioid peptide ligands were also detected in human DRG with their respective Ct values (Ct value range: 20–34, see Table 1) (Figure 6). Most interestingly, PENK (Ct value 25) expression in human DRG neurons was the highest (100-fold) among the opioid peptides, whereas POMC (Ct value 20) expression was highest in rat DRG neurons (Figure 6).

### 3.5. Expression of Pain-Relevant mRNA Transcripts in Relation to MOR Transcripts in Human and Rat DRG

**Figure 7:**
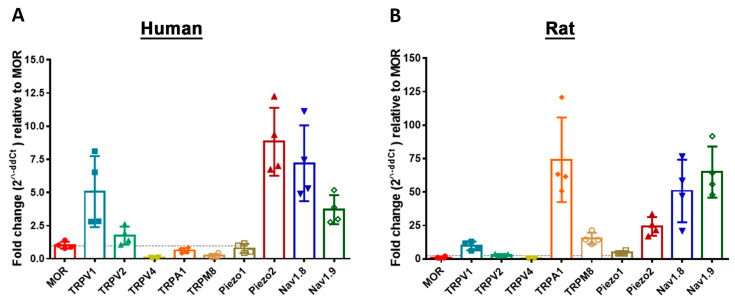
Detection of MOR mRNA transcripts in relation to key pain-signaling molecules in human (**A**) versus rat (**B**) sensory DRG neurons. In using specific mRNA primers, quantitative real-time PCR showed the expression of key pain-signaling-molecule mRNAs in relation to MOR mRNA transcripts of both human (**A**) and rat (**B**) sensory DRG neurons. Moreover, quantitative real-time PCR analyses of human DRG neurons revealed that Piezo2 mRNA was more abundant than that of other key pain-signaling molecules, whereas TRPA1 mRNA was superior in rat DRG neurons (**A**). Data are expressed as means ± SD. ΔCt values were obtained by Ct_gene_–Ct_18S_ housekeeping gene and subsequently related to ΔCt values of MOR according to the ΔΔCt method [24].

In these real-time PCR experiments, we determined the expression levels of key pain-signaling molecules relative to those of the pain-modulating opioid receptor MOR in both human and rat sensory DRG neurons. Since the analysis was performed within the same sample, potential confounding factors were consistent and thus effectively controlled for. The results revealed that TRPV1, TRPV2, Piezo2, Nav1.8, and Nav1.9 were expressed at levels 2- to 8-fold higher than MOR in human DRG (Figure 7).

In contrast, TRPV4, TRPA1, TRPM8, and Piezo1 exhibited a slightly lower expression than MOR. In naïve rat DRG, TRPA1 and TRPM8 showed the highest mRNA expression, followed by Piezo2, Nav1.8, and Nav1.9, alongside TRPV1, TRPV2, and Piezo1 transcripts; however, the differences in expression levels of key pain-signaling molecules relative to MOR were markedly greater compared to those observed in human DRG (Figure 7).

### 3.6. Inhibition of VDCC Activity by the MOR Selective Agonist Fentanyl in Rat DRG Neurons

**Figure 8:**
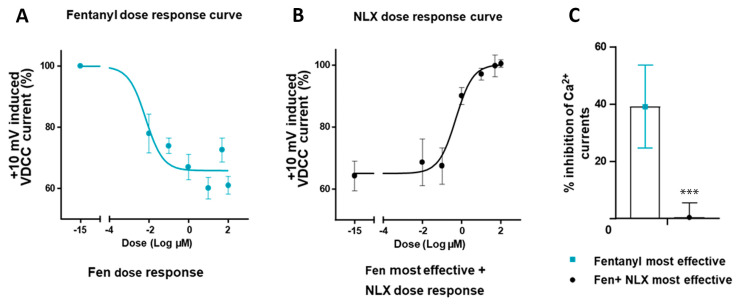
Inhibition of voltage-dependent calcium currents by the MOR agonist fentanyl in rat DRG neurons. Whole-cell patch-clamp recordings in isolated rat DRG neurons were used to assess fentanyl’s dose-dependent inhibition of +10 mV-evoked inward calcium currents (**A**). This inhibition was reversed in a dose-dependent manner by the MOR antagonist naloxone (**B**). The most effective fentanyl dose reduced calcium currents by approximately 40%, and naloxone completely abolished this effect, confirming MOR specificity on VDCCs (**C**). *** *p* < 0.001 comparison of the most effective fentanyl-induced inhibition vs. fentanyl + naloxone-treated DRG neurons (n = 16–20); two-tailed *t*-test. Data are presented as means ± SEM.

Using the whole-cell patch-clamp configuration, depolarization of rat DRG neurons (n = 16–20, Ø between 20–30 µm) from a holding potential of −80 mV to +10 mV (100 ms) elicited the activation of VDCCs, resulting in inward Ca^2+^ currents. Fentanyl, a potent and selective MOR agonist, significantly inhibited these depolarization-induced calcium currents in a dose-dependent manner (Log IC_50_ = −7.95), consistent with an opioid-receptor-mediated mechanism (Figure 8A).

The fentanyl-induced inhibition of VDCC currents reached a maximum of approximately 40% and was completely reversed by the MOR antagonist naloxone, further confirming the specificity of the effect to MOR activation (Figure 8).

## 4. Discussion

Since the first clinical report on the peripheral analgesic effects of intra-articularly applied morphine in patients undergoing knee surgery [6], evidence supporting the presence of opioid receptors on peripheral sensory nerve terminals and the analgesic efficacy of their locally applied agonists was initially met with skepticism [7,8,9]. However, accumulating evidence has confirmed the existence of opioid receptors on peripheral sensory neurons in animals [4].

In this study, we report the detection of mRNA transcripts, immunoreactivity, and localization of key pain-signaling molecules (TRPV1, TRPV4, TRPA1, Piezo1, Piezo2, and Nav1.8) in human DRG, along with the three main opioid receptors (MOR, DOR, and KOR) and the precursor peptides of their endogenous ligands (POMC, PENK, and PDYN). Notably, these opioid receptors were identified using specific antibodies in a subpopulation of human CGRP-IR sensory neurons, which also exhibited the expression of well-established pain-signaling molecules. These include the heat- and proton-sensitive pain transduction receptors TRPV1 and TRPV4, the cold-sensitive receptor TRPA1, the mechanosensitive ion channels PIEZO1 and PIEZO2, and the TTX-resistant sodium channel Nav1.8, which is exclusively expressed in peripheral nociceptive neurons. Our findings corroborate recent excellent studies on human DRG [20,21,22] by providing additional imunohistochemical evidence of the expression of mu (MOR), delta (DOR), and kappa (KOR) opioid receptors, opioid peptide precursors (POMC, PENK, and PDYN), and key pain-signaling molecules in human compared to rat DRG. Moreover, the functional relevance of this opioidergic system was confirmed in the present study through the MOR-specific inhibition of depolarization-induced VDCC calcium currents. Taken together, our results suggest that MOR is the predominant opioid receptor in human nociceptive DRG neurons, while PENK is the most prominent opioid receptor ligand precursor. This suggests that the transduction and transmission of painful stimuli may already be modulated at the level of peripheral sensory neurons by opioid receptor agonists [34,35], expanding their therapeutic potential beyond the central nervous system toward a more peripherally targeted approach, thereby reducing the risk of feared central side effects.

Human DRG have recently been the focus of intensive investigation, primarily through transcriptomic and proteomic analyses of large sets of gene and protein products [18,36,37,38,39,40,41,42]. However, few studies have examined opioid receptor subtypes and functions in human DRG using electrophysiological and multiplex in situ-hybridization approaches [20]. As an extension of these studies, we first aimed to determine by immunofluorescence microscopy whether key pain-signaling molecules are detectable in human DRG. Consistent with our previous findings [34,43,44] and those of other studies in rodents [45,46,47], we identified the heat-sensitive TRPV1, the mechano-sensitive TRPV4, and the cold-sensitive TRPA1 in human DRG neurons. These receptors co-localized with CGRP, a sensory neuron marker predominantly associated with peptidergic DRG neurons. Interestingly, TRPV4 staining occurred not only in the cytoplasmic area but also in the nuclear area, which was first described by Espadas-Álvarez et al. [48]. Similarly, we detected the mechanosensitive ion channels PIEZO1 and PIEZO2, as well as the TTX-resistant sodium channel Nav1.8, all of which co-localized with CGRP in human DRG neurons. Using specific antibodies, we identified all three major opioid receptors (MOR, DOR, and KOR) in human DRG neurons, also with a predominant colocalization with CGRP.

To further confirm the expression of the clinically relevant MOR in human peripheral nociceptive DRG neurons, we demonstrated its colocalization with the TTX-resistant sodium channel Nav1.8, which is exclusively expressed in peripheral nociceptive neurons [49]. This finding aligns with the results of previous functional studies in conditional MOR (Nav1.8-Cre x Oprm1-/-) [50] or conditional DOR (Nav1.8-Cre x Oprd1fl/fl) knockout mice [51]. In these models, the deletion of MOR or DOR from Nav1.8-positive DRG neurons led to a significant reduction in MOR- or DOR-mediated analgesia under inflammatory and neuropathic pain conditions. Correspondingly, MOR-agonist-mediated G-protein coupling in DRG neurons was also significantly attenuated [50].

It was particularly intriguing to determine whether the precursor peptides of endogenous opioid ligands (POMC, PENK, and PDYN) were also detectable in sensory DRG neurons using immunofluorescence microscopy. Indeed, both POMC and PENK were identified in a subset of DRG neurons, whereas PDYN immunoreactivity was only sparsely detected. To date, only one study in rodents has reported POMC gene expression in mouse sensory trigeminal ganglion neurons, along with POMC-immunoreactive nerve fibers in the dura mater of mouse meninges [52]. Another rodent study [53] detected PENK and PDYN mRNA in lumbar DRG using in situ hybridization, particularly following sciatic nerve transection, which resulted in the upregulation of PDYN mRNA and downregulation of PENK mRNA. While the expression and role of PENK and PDYN have been extensively studied in the spinal cord [54,55], opioid peptide expression in sensory DRG neurons has received considerably less attention.

We then sought to quantitatively assess the mRNA expression of MOR, DOR, and KOR, as well as their corresponding endogenous ligand precursors (POMC, PENK, and PDYN) in human DRG neurons. All three opioid-receptor mRNAs were detected with moderate Ct values ranging between 25 and 30, with MOR expression being five times higher than that of DOR and KOR in the DRG of young adults.

These findings align with the results of both an earlier study [56] and a more recent study [16], which identified MOR and DOR transcripts via in situ hybridization in immunohistochemically stained NF200-positive myelinated neurons, IB4- or P2X3R-positive small nonpeptidergic neurons, and Tac1-positive peptidergic neurons in rats and mice. Notably, mRNA transcripts for all three precursor peptides (POMC, PENK, and PDYN) were also detected in human DRG. PENK mRNA exhibited the highest expression, being 100-fold higher than POMC mRNA in human DRG, whereas POMC mRNA expression was most prominent in rat DRG. This finding suggests that, in addition to the descending inhibitory neurons from the RVM [57,58] and the local inhibitory interneurons of the spinal dorsal horn [54], incoming peripheral sensory neurons may themselves contribute to the modulation of painful stimuli.

We then analyzed MOR expression in relation to the mRNA expression of well-known pain-signaling molecules and found that TRPV1, TRPV2, Piezo2, Nav1.8, and Nav1.9 were expressed at levels 2- to 8-fold higher than MOR in human DRG of young adults. In contrast, TRPV4, TRPA1, TRPM8, and Piezo1 exhibited a somewhat lower expression than MOR. In comparison, TRPA1 and TRPM8 showed the highest mRNA expression levels, alongside TRPV1, TRPV2, Piezo2, Nav1.8, and Nav1.9 in naïve rat DRG. However, these expression profiles are likely to vary based on factors such as sex, age, and the presence of painful and/or non-painful comorbidities [15,37]. Encouragingly, much more extensive and thoroughly investigated studies on these key pain-signaling molecules have been recently carried out using RNAscope in situ hybridization [15,16,17,18,19]. To elucidate the functional relevance of MOR in DRG neurons, we demonstrated that in acutely dissociated rat DRG neurons, depolarization-triggered inward Ca^2+^ currents of VDCCs were inhibited by the potent and selective MOR agonist fentanyl in a concentration-dependent manner. Furthermore, this significant inhibition of Ca^2+^ currents was dose-dependently reversed by the opioid receptor antagonist naloxone. These findings are consistent with those of previous studies in rats [59,60] and were further supported by more recent research in human DRG neurons [20], which functionally characterized all three opioid receptors (MOR, DOR, and KOR) and confirmed their ability to inhibit voltage-gated Ca^2+^ currents.

What do these findings mean for the analgesic efficacy of peripherally applied opioids? Following initial reports about the peripheral analgesic effects of opioids [61,62], there is compelling evidence that low, systemically inactive doses of MOR agonists can induce significant analgesia [4]. However, compared to systemically administered opioids, the analgesic effects of peripherally applied opioids are constrained by a limited dose range, often exhibit a ceiling effect, and are therefore significantly inferior to the systemic application of opioids. This is because systemic opioids activate MOR not only at the peripheral level but also at the spinal and supraspinal levels, leading to more robust analgesia [1]. The only viable approach to overcoming this limitation is the development of peripherally restricted opioids. Unfortunately, despite extensive research, this strategy has not yet achieved sufficient clinical success [10,11,63]. In this context, it is noteworthy that systematic literature reviews and meta-analyses have failed to conclusively demonstrate a clear benefit of locally applied opioids in patients undergoing knee surgery, aside from small-to-moderate effects in some cases [7,9,64]. Future innovative strategies may provide new perspectives on the development of peripherally restricted opioid compounds, such as those designed to selectively activate in the acidic inflammatory milieu while sparing the central nervous system, thereby minimizing systemic side effects [65].

This study demonstrates structural and transcriptional evidence of the opioid receptors MOR, DOR, and KOR, along with the corresponding precursor peptides of their endogenous ligands (POMC, PENK, and PDYN) in human DRG, in comparison to rat DRG. Our findings demonstrate that MOR is the predominant opioid receptor, while PENK is the most prominent endogenous ligand precursor in human DRG. Opioid receptors were found to co-localize within the same subpopulation of CGRP-immunoreactive sensory DRG neurons that also express key pain-signaling molecules, including the heat- and proton-sensitive pain transduction receptors TRPV1 and TRPV4, the cold-sensitive receptor TRPA1, the mechanosensitive ion channels PIEZO1 and PIEZO2, and the TTX-resistant sodium channel Nav1.8, which is exclusively expressed in peripheral nociceptive neurons. These findings align with studies in rat DRG, where the functional relevance of opioid receptors was demonstrated through the MOR-specific inhibition of depolarization-induced VDCC calcium currents.

This study has several limitations. Notably, only two human samples (one male and one female) were analyzed, which limited the ability to carry out reliable quantifications of specific DRG neuron subtypes and hindered meaningful comparisons with rat DRG data. Although there is an approximately 95% overlap in the amino acid sequences of MOR, DOR, and KOR between rats and humans, the specificity of our antibodies has not been thoroughly validated yet and could therefore be hampered. Moreover, the fixation process of our commercially obtained human DRG tissue was out of our hands and may have impaired the specificity of the antibodies. Rat DRG tissue samples were obtained only from male rats versus one male and one female human DRG. Finally, a comprehensive analysis of the distribution of MOR, DOR, and KOR in human DRG should be conducted on more samples and in a much more thorough way. The aim of this study was to perform a first immunohistochemical detection of MOR, DOR and KOR in human DRG together with their opioid peptide precursors compared to rat DRG neurons. Since our human tissue samples came from two young fatal-accident victims, the results could also differ considerably depending on the age, history of pain, medication use, etc. of the humans.

## 5. Conclusions

Collectively, these results expand the therapeutic potential of opioid analgesics beyond their traditionally recognized central mechanisms of action. By targeting peripherally accessible opioid receptors on primary afferent neurons, peripherally restricted opioid drugs may offer effective analgesia while minimizing the central side effects typically associated with systemic opioid use.

## Figures and Tables

**Table 1:** Comparison of respective Ct values (median, range) from human versus rat DRG determined by real-time PCR.

Gen	Human_DRG	Rat_DRG
MOR	26	25
DOR	28	30
KOR	28	26
POMC	32	20
PENK	25	24
PDYN	34	30
TRPV1	23	22
TRPV4	29	26
TRPA1	26	19
TRPM8	28	21
Piezo1	26	23
Piezo2	22	20
Nav1.8	22	20
Nav1.9	23	19

## Data Availability

Data can be accessed upon request by contacting the first author via e-mail: shaaban.mousa@charite.de.

## Supplementary Materials

The following supporting information can be downloaded at: https://www.mdpi.com/article/10.3390/cells14100694/s1, Table S1: List of Primers for Taqman RT-PCR of human DRG; Table S2: List of Primers for Taqman RT-PCR of rat DRG; Table S3: Table of Primary Antibodies used; Figure S1: Immunofluorescence staining of human dorsal root ganglia tissue using Alexa Fluor 594 donkey anti-rabbit antibody (Texas red immunofluorescence) and Alexa Fluor 488 goat anti-mouse antibody (FITC green fluorescence) as secondary antibodies with omission of the respective primary antibodies (blank control). Nuclei were counterstained with 4′,6-diamidino-2-phenylindole (DAPI; bright blue). Scale bar = 40 µm.
