# Supplementary material for: The Identification of Opioid Receptors and Peptide Precursors in Human DRG Neurons Expressing Pain-Signaling Molecules Confirms Their Potential as Analgesic Targets
Source: Cells. 2025 May 11;14(10):694. doi: 10.3390/cells14100694 (PMC12110618; doi:10.3390/cells14100694)

Table S1: List of Primers for Taqman RT-PCR of human DRG

Gene	Access. Nr.	Forward	Reverse
**MOR**	NM_000914.4	5'-acaggcaaggttccatagattgt-3'	5'-gcactggcataatgaaggcg-3'
**DOR**	NM_000911.3	5'-ggcatcgtccggtacact-3'	5'-caggtacttggcactctggaa-3'
**KOR**	NM_000912.4	5'-cgtgatcatccgatacacaaaga-3'	5'-gaccgtactctgaaagggca-3'
**OPRL1**	NM_000913.6	5'-ctggggaactgccttgtcat-3'	5'-gtcagcaggaccagagtgtc-3'
**POMC**	NM_000939.3	5'- caagcgctcctactccatgg-3'	5'-cagtcagctccctcttgaactc-3'
**PENK**	NM_001135690.2	5'-cttcctggcttgcgtaatgg-3'	5'-tggctttcttccggtttgct-3'
**PDYN**	NM_001190892.1	5'-gagtctgagctgatgagggatg-3'	5'-agcgtttgtacaggtcctcatg-3'
**TRPV1**	NM_080704.4	5'-gcaggacaagtgggacagat-3'	5'-tcttaaagggaggcaagcca-3'
**TRPV2**	NM_016113.5	5'-aaactgctgcaggcgaaatg-3'	5'-ggcggcctgcttcttca-3'
**TRPV4**	NM_021625.5	5'-tcaaagtcttcaaccggcct-3'	5'-ccccgtagatggctctcgaa-3'
**TRPA1**	NM_007332.3	5'-ggggccctggttctgtaaat-3'	5'-atacgcccataactggctgc-3'
**TRPM8**	NM_024080.5	5'-aaactggttgcgaacttccg-3'	5'-taggagacacgtcgtggagt-3'
**PIEZO1**	NM_001142864.4	5'-aagaacatgctgtcgctcct-3'	5'-acggtgcatacaaggctgaa-3'
**PIEZO2**	NM_001378183.1	5'-tggtacgcaacccattaccc-3'	5'-agtcaccagcaggccatcag-3'
**Nav.1.8**	NM_006514.4	5'ctccgtccttgaggaactcg-3	5'-tctgcaaagggatccgtcac-3'
**Nav.1.9**	NM_001349253.2	5'agccaggaatctcgggtgaa-3	5'-agccagagagtcggaagtga-3'
**S18**	NR_046237	5'-cggctaccacatccaaggaa-3'	5'-cctggaattaccgcggct-3'

Table S2: List of Primers for Taqman RT-PCR of rat DRG

Gene	Access. Nr.	Forward	Reverse
**MOR**	NM_001038597	5’-ttacggcctgatgatcttacga-3’	5’-ggtgatcctgcgcagattc-3’
**DOR**	NM_012617	5’-gctgggctacgccaacag-3’	5’-cggaagcagcgcttgaag-3’
**KOR**	NM_017167	5’-tctttatcctggtcgaggctcta-3’	5’-cccaaggcaatgcagaagtaa-3’
**POMC**	NM_139326.2	5’- agagttcaagagggagctggaa-3’	5’-gtcggccttctcggtatcc-3’
**PENK**	NM_017139.1	5’-tccgacctgctgaaagagcta-3’	5’-tgctttcctgttggtggctat-3’
**PDYN**	NM_019374.3	5’-aagcttaagtgggacaaccagaaa-3’	5’-gttctcctgggaccgagtca-3’
**TRPV1**	NM_031982	5’-agtgagacccctaaccgtca-3’	5’-cggaaatagtccccaacggt-3’
**TRPV4**	NM_023970.1	5’-gcccatggattcgttgttcg-3’	5’-tgtggctgcttctctacgac-3’
**TRPA1**	NM_207608.1	5’-tggggaaagacatggactgc-3’	5’-acattcagcgcttcacagga-3’
**TRPM8**	NM_134371.3	5’-gcagtggtacatgaacggagt-3’	5’-tgaagagtgaagccggaatac-3’
**PIEZO1**	NM_001077200	5’-ttccttttccaggggttccg-3’	5’-cgtggtgtcagtccataccc-3’
**PIEZO2**	XM_039097392	5` ggcctaggctccgtaaagtg 3`	5` ccgtagttccttggcgtgaa 3`
**Nav.1.8**	NM_017247.1	5’-cacggatgacaacaggtcac-3’	5’- gatcccgtcaggaaatgaga-3’
**Nav.1.9**	NM_019265.2	5’ccaggagccttgtttcccat-3	5’-tttatgcacagccactgagg-3’
**18S**	NR_046237.2	5’cggctaccacatccaaggaa-3	5’-gctggaattaccgcggct-3’

Table S3: Table of Primary Antibodies used

Antigen	Immunogen	Manufacturer, Species, Type, Catalogue Number	Dilution used
**Mu opioid receptor**	a synthetic peptide, aa 386–398 from C-terminus of MOR	a gift from S. Schulz and V. Höllt (Magdeburg, Germany), rabbit polyclonal, #Schulz et al., 1997; [28]	1:1,000
**Kappa opioid receptor**	a synthetic peptide, aa 384–398 from N-terminus of KOR	a gift from S.J. Watson (Michigan, USA), rabbit polyclonal, # Mansour et al., 1996; [29]	1:1,000
**Delta opioid receptor**	a synthetic peptide, aa 3–17 of the murine receptor 384–398 from N-terminus of DOR	a gift from R. Elde (Minneapolis, MN, USA), rabbit polyclonal, # Riedl et al., 2009; [27]	1:2,000
**TRPV1**	raised against C-terminus of the TRPV1 receptor (GSLKPEDAEVFKDSMVPGEK)	Neuromics (Edina, CA, USA), guinea pig polyclonal, Shaqura et al., 2014; [43]	1:1.000
**TRPV4**	raised against intracellular C-terminus of the TRPV4 receptor (CDGHQQGYAPKWRAEDAPL)	alomone labs (Jerusalem, Israel), rabbit polyclonal, # ACC-034	1:5.00
**CGRP**	synthetic entire calcitonin gene-related peptide	Peninsula Laboratories (CA, USA), guinea pig polyclonal, # T-5027	1:1,000
**CGRP**	raised against entire calcitonin gene-related peptide	Santa Cruz Biotechnology, Inc. (Texas, USA), mouse monoclonal, # SC-57053	1:500
**Na$_V$1.8**	Fusion protein amino acids 1724-1956 (cytoplasmic C-terminus) of rat Nav1.8	NeuroMab (University of California, Davis/NIH), mouse monoclonal, Cat # 75-166, RRID: AB_2183861	1:200
**PENK**	peptide PENK A, 121–134 of human PENK	SphingoTec GmbH (Hennigsdorf, Germany), mouse monoclonal, Cat # 10-30-106, Ernst et al., 2006	1:250
**POMC**	N-terminal amino acids 1–50 of POMC,	Biogenesis Ltd (Poole, UK), mouse monoclonal, Cat # 10-30-106, Sitte et al., 2007	1:250
**PDYN**	residues 235-248 of the rat proDynorphin	Neuromics Antibodies (MN, USA), guinea pig, Cat # GP10110, Gardell et al., 2003	1:250
**TRPA1**	a synthetic peptide from rat TRPA1 conjugated to blue carrier protein	Novus Biologicals (CO, USA), rabbit polyclonal, Cat # NB100-98841, Yu et al., 2009	1:1000

**PIEZO1**	internal portion of the human PIEZO1 protein (between residues 1300-1350)	Novus Biologicals (CO, USA), rabbit polyclonal, Cat # NBP1-78537, Matsunaga et al., 2021	1:500
**PIEZO2**	internal portion of the human PIEZO2 protein (between residues 1450-1500)	Novus Biologicals (CO, USA), rabbit polyclonal, Cat # NBP1-78538, Lee et al., 2014	1:500

Reference:

27. Riedl, M.S.; Schnell, S.A.; Overland, A.C.; Chabot-Doré, A.J.; Taylor, A.M.; Ribeiro-da-Silva, A.; Elde, R.P.; Wilcox, G.L.; Stone, L.S. Coexpression of alpha 2A-adrenergic and delta-opioid receptors in substance P-containing terminals in rat dorsal horn. *The Journal of comparative neurology* **2009**, *513*, 385-398, doi:10.1002/cne.21982.
28. Schulz, S.; Schreff, M.; Koch, T.; Zimprich, A.; Gramsch, C.; Elde, R.; Höllt, V. Immunolocalization of two mu-opioid receptor isoforms (MOR1 and MOR1B) in the rat central nervous system. *Neuroscience* **1998**, *82*, 613-622, doi:10.1016/s0306-4522(97)00288-1.
29. Mansour, A.; Burke, S.; Pavlic, R.J.; Akil, H.; Watson, S.J. Immunohistochemical localization of the cloned kappa 1 receptor in the rat CNS and pituitary. *Neuroscience* **1996**, *71*, 671-690, doi:10.1016/0306-4522(95)00464-5.
43. Shaqura, M.; Khalefa, B.I.; Shakibaei, M.; Zöllner, C.; Al-Khrasani, M.; Fürst, S.; Schäfer, M.; Mousa, S.A. New insights into mechanisms of opioid inhibitory effects on capsaicin-induced TRPV1 activity during painful diabetic neuropathy. *Neuropharmacology* **2014**, *85*, 142-150, doi:10.1016/j.neuropharm.2014.05.026.

Supplemental Figure S1:
Immunofluorescence staining of human dorsal root ganglia tissue using Alexa Fluor 594 donkey anti-rabbit antibody (Texas red immunofluorescence) and Alexa Fluor 488 goat anti-mouse antibody (FITC green fluorescence) as secondary antibodies with omission of the respective primary antibodies (blank control). Nuclei were counterstained with 4′,6-diamidino-2-phenylindole (DAPI; bright blue). Scale bar = 40 µm.

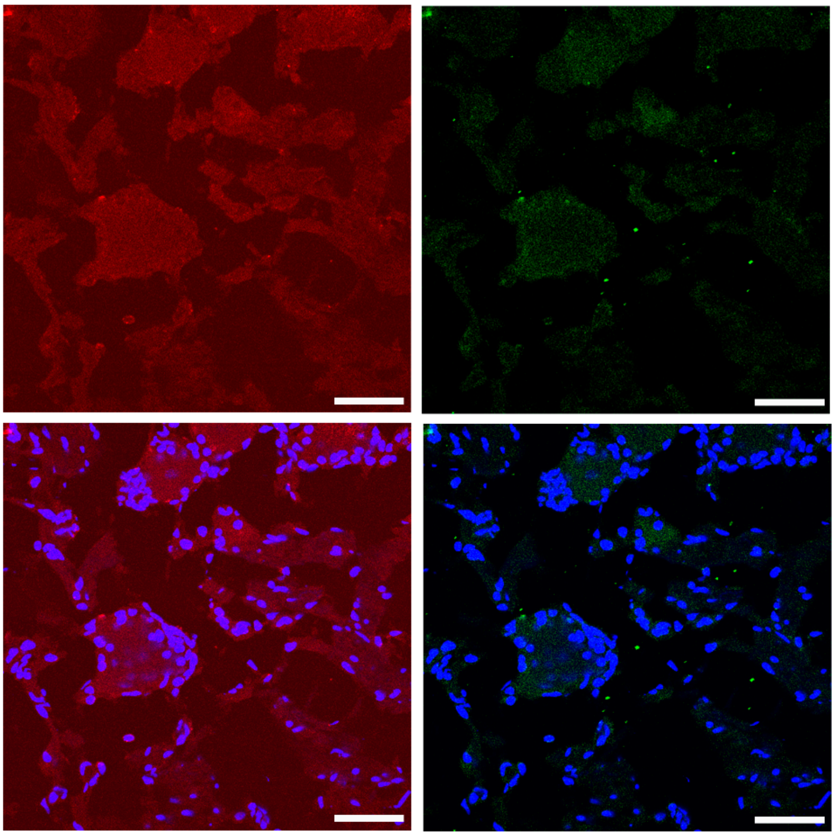

Supplement: Supplementary file 1 [file cells-14-00694-s001.zip › cells-3556250-supplementary.pdf]
